# Modeling Sorption of Hydrocarbons in Polyethylene with the SAFT-γ Mie Approach Combined with a Statistical-Mechanical Model to Describe Semicrystalline Polymers

**DOI:** 10.1021/acs.macromol.3c01336

**Published:** 2023-12-04

**Authors:** Michele Valsecchi, Amparo Galindo, George Jackson

**Affiliations:** † Department of Materials, Imperial College London, South Kensington Campus, London SW7 2AZ, U.K.; ‡ Department of Chemical Engineering, Imperial College London, South Kensington Campus, London SW7 2AZ, U.K.

## Abstract

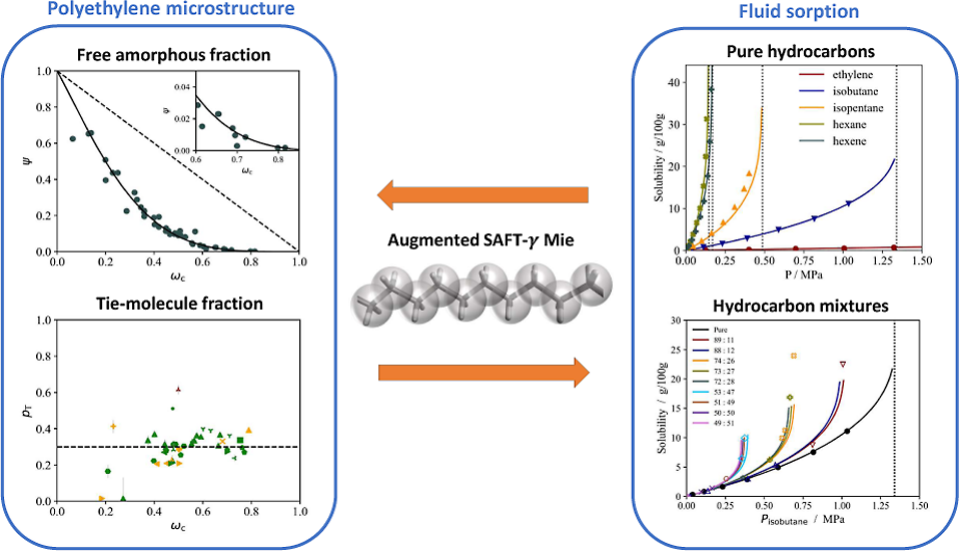

A recently developed statistical-mechanical model is applied systematically to estimate the fraction of tie-molecules (polymer chains linking different crystals directly or via entanglements) in semicrystalline polyethylene (PE) samples. The amorphous domains of the polymer are divided into constrained interlamellar domains and “free” outer-lamellar domains. A set of model parameters is assigned to each sample by correlating previous experimental measurements and minimizing the difference between the predicted solubility of pure hydrocarbons in the sample and the experimental values. We show that the sorption isotherms of multiple pure fluids in each sample can be described by a single parameter set, proving that the polymer–solute interactions (described accurately by the SAFT-γ Mie EoS) are decoupled from the sample-specific properties of the polymer. We find that ∼30% of the crystalline stems in the lamellae of PE are connected to tie-molecules, within the bounds suggested by previous theoretical and computational work. The transferability of the sample-specific parameters is assessed by predicting cosolubility effects and solubility at different temperatures, leading to good agreement with experimental data.

## 1 Introduction

Semicrystalline polymers are ubiquitous in modern society. Around 400 million tonnes of plastics are produced each year, with semicrystalline polyethylene (PE) alone accounting for about a third of the total production.^1^ One of the qualities for which these materials are most valued is their excellent barrier performance with respect to the solubility and diffusivity of gases and liquids. Around 40% of the total plastic production is destined for packaging,^2^ the purpose of which is protecting its content from the external environment by preventing fluid permeation. Semicrystalline polymers like high-density PE, polyvinyl chloride, polyamide, polyether ether ketone, polytetrafluoroethylene, and polyvinylidine fluoride are also used extensively for fluid transport either as pipe materials^3,4^ or as liners to protect metal surfaces from corrosion and embrittlement.^5,6^

In some applications, promoting fluid solubility in semicrystalline polymers can be desirable. During PE production, greater concentrations of ethylene in the growing polymer grains near the catalyst sites increases the reaction rate and yield.^7,8^ In the detergent and perfume industry, ensuring the persistence of certain ingredients in hair or fibers can lead to longer lasting freshness and softness.^9,10^ Perhaps more importantly, the ability of oxidizing agents to diffuse within a semicrystalline polymer is one of the factors affecting the degradation rate when left in the environment.^11−13^ This is particularly relevant today as the sheer amount of synthetic plastic produced combined with its short usage lifespan^2^ and long degradation times^14^ has led to serious concerns of the resulting environmental impact.

Predicting the gas and liquid solubility in semicrystalline polymers is therefore crucial for optimizing the production, performance, and degradation behavior. The thermodynamic and transport properties of these materials can vary greatly depending on microstructural features such as the degree of crystallinity^15,16^ and the connectivity of the crystalline lamellae via tie-molecules.^17−19^ These can be functions of a number of system- and process-dependent factors such as the polymer’s molecular weight distribution,^20,21^ the presence of branches on the polymer molecules,^22,23^ or the cooling history.^18,24^ A complete understanding on how all these factors determine the “state” of a semicrystalline polymers is however still lacking,^19,25^ partly due to the nonequilibrium nature of these systems. At the same time, the direct measurement of certain sample-specific properties such as the average lamellar spacing or the density of tie-molecules requires advanced characterization techniques (e.g., small-angle X-ray^26,27^ or neutron^24,28^ scattering for the former) or is currently unachievable.

Statistical thermodynamic models can help shed light on the molecular mechanisms that govern the physics of these systems and provide a link between their microstructural features and macroscopic thermodynamic observables such as the elastic moduli^29−32^ and sorption behavior.^17,18,33^ These models can, on one hand, provide estimates of a sample’s microscopic features utilizing data from simple experiments (e.g., tensile tests and swelling measurements), while on the other, guide design and production by allowing one to find the optimal set of microscopic properties for each application. In the current work, a recent model^33^ describing sorption in semicrystalline polymers is benchmarked against a large set of experimental data for the solubility of hydrocarbon fluids in semicrystalline PE samples. This prototypical polymer is chosen due to the simplicity of its repeating unit and the large amount of PE data available. The optimal model parameters, in particular, the tie-molecule fraction *p*_T_, are estimated for each sample by minimizing the difference between the experimental data and the theoretical predictions. The trends in the optimal values of *p*_T_ are then critically assessed to gauge the ability of the theory to describe the physics of semicrystalline polymers. Finally, the robustness of our methodology is showcased by predicting the solubility at different temperature and the cosolubility effects in a subset of the samples analyzed.

## 2 Methods

### 2.1 Model Highlights

In this section, the main features of the model used are outlined after providing a brief survey of experimental evidence and previous modeling approaches. The reader is referred to the original work^33^ for an in-depth discussion.

Semicrystalline polymers can be characterized by their crystallinity ω_c_, defined as the ratio between the mass of crystalline domains and the total polymer mass. This quantity can be determined indirectly by measuring the density of a semicrystalline sample,^20,33^ the melting curves (via differential scanning calorimetry (DSC)^34−36^), or the diffraction pattern in SAXS experiments^37,38^ and then comparing the results to reference values for the “pure” amorphous and/or crystalline domains obtained via correlation of experimental data or theoretical considerations. For example, the knowledge of the specific volume of the polymer (*v*) at a given temperature T* allows one to calculate the crystallinity if the specific volumes of the amorphous (*v*_a_) and crystalline (*v*_c_) domains at the same temperature T* are known
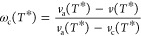
1

Similarly, if the specific melting enthalpy Δ*h*_m_ (measured via DSC) is reported, the crystallinity can be calculated using
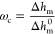
2where Δ*h*_m_^0^ is the reference melting enthalpy of a perfect polymer crystal. Clearly, in these calculations one assumes that both the amorphous and crystalline domains are homogeneous and can be assigned a unique value for the respective intensive property (i.e., specific volume or enthalpy). These assumptions highlight the fact that different measurements of crystallinity can sometimes yield different results,^35^ making it a somewhat ill-defined quantity.

Despite this ambiguity, crystallinity is the main feature influencing the solubility of a small molecule in semicrystalline polymers, as was first hypothesized by Richards^15^ and later shown by Michaels and Bixler.^16,39^ Hereafter, the solubility *S*_*i*_ of a substance *i* in a polymer sample is intended as the ratio of the mass *m*_S,*i*_ of solute dissolved in the polymer and the total polymer mass *m*_p_. Michaels and Bixler postulated that the crystalline domains are impermeable to solutes and that the amorphous domains can be treated effectively as subcooled polymer liquids. If a *N*-component fluid at temperature *T*, pressure *P*, and composition ***y*** is in contact with a semicrystalline polymer, Michaels and Bixler’s hypothesis corresponds to
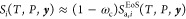
3where *S*_a,*i*_^EoS^ is the solubility of component *i* in a subcooled polymer liquid and the superscript EoS indicates that the function varies depending on the choice of equation of state (or, more generally, theory of liquid polymer mixtures). Equation 3 is the first example of decoupling the polymer–solute interaction (described by the equation of state and its parameters) from polymer sample-specific properties (the sample’s crystallinity ω_c_). Furthermore, the Michaels and Bixler model embodies the “two domain” picture employed by all of the prominent solubility models developed to date,^17,18,40−44^ with the exception of our recent work.^33^ According to this picture, the amorphous domains are assumed to be homogeneous and all interfacial regions are neglected.

It is generally accepted that solutes do not dissolve in the crystalline domains of most semicrystalline polymers. Heuristically, this is expected due to the high enthalpy of the formation of defects in the crystal lattice. At the same time, assuming that the amorphous domains are in effect liquid polymer is justified by the disordered, “liquid-like” nature of the polymer in these regions.^45^ However, Rogers et al.^17^ and later Michaels and Hausslein (MH)^18^ questioned this notion by showing that the solubility *S*_*i*_ is lower than the one predicted using eq 3. The authors justified this finding by arguing that tie-molecules (i.e., polymer segments linking two different crystals directly or indirectly via entanglements) decrease their conformational entropy upon swelling, a concept that was previously employed to justify the solubility reduction in swollen elastomers (e.g., the Flory–Rehner theory^46^).

Rogers et al. and subsequent authors^28,40,41,47^ simply modified the Flory–Rehner theory to account for the structural differences between elastomers and the amorphous domains of semicrystalline polymers. However, in the past decades, an extensive body of research has shown that in “crystal-mobile” polymers such as PE, isotactic PP, and PEO,^48−50^ the longitudinal mobility of polymer chains in the crystals (the so-called α-relaxation^51,52^) causes the amorphous chains to be locally in equilibrium with the crystalline domains with respect to exchanges of monomers, as demonstrated by reversible partial melting of the lamellae.^26,27,29,30,33,53,54^ Michaels and Hausslein realized that this phenomenon makes the tension of the tie-molecules, and therefore the excess “elastic” activity of solutes dissolved, temperature-dependent, in line with their experimental findings and in contrast with the Flory–Rehner theory.

Despite the success of the MH theory in describing the solubility of various organic compounds in PE,^37,47,55−60^ the original MH model has two major issues. The model (like the Flory–Rehner theory^17,40,41,46^) makes inconsistent use of the Gaussian approximation for the tie-molecules’ end-to-end probability distribution. This approximation is in fact only valid for small to moderate chain stretching, whereas the local-equilibrium hypothesis predicts that amorphous chain segments should be fairly taut at temperatures sufficiently lower than the melting point^29,30,33^ (e.g., room temperature for PE). Furthermore, the original model assumes isotropic swelling of the amorphous domains^18^ despite the markedly one-dimensional character of swelling in the interlamellar domains.^24,28,40,41^

In our recent work,^33^ we showed that the inclusion of a realistic end-to-end distribution accounting for finite-chain extensibility (i.e., the Langevin approximation) and the replacement of isotropic swelling with one-dimensional swelling is necessary to avoid unphysical predictions for the relative extension of the chain-segments and capture the correct temperature dependence of the excess activity. Moreover, our model is the first in the context of solubility models to relax the two-domain picture. The amorphous domains are divided into a constrained portion with a high tie-molecule density (corresponding to the interlamellar domains) and an unconstrained, mobile portion (corresponding to the “free” amorphous domains, presumably polymer outside of the lamellar stacks) with a total mass fraction ψ that behaves approximately as a subcooled polymer liquid. This “three-domain” picture (crystalline domains plus two types of amorphous domains) has been proposed recently^61^ to explain the bimodal nature of the relaxation times of amorphous chains in ^1^H NMR experiments, and is necessary to explain the increase of solubility of simple fluids near their vapor pressure,^33^ as swelling in the interlamellar domains is severely restricted due to the high density of tie-molecules.^24,62^ Note that the interfacial regions are neglected in our model.

Accounting for two types of amorphous domains means that a three-phase equilibrium has to be solved when an external fluid is in contact with a semicrystalline polymer. At fixed temperature *T* and pressure *P* and composition ***y***, if **μ**_s_(*T*, *P*, ***y***) is the vector of chemical potentials of *N* solutes imposed by an external fluid of infinite extension, the solubilities of the solutes in the free (***S***_a_^F^) and interlamellar (***S***_a_^IL^) amorphous domains are found by solving the equations
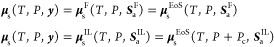
4

As before, the superscript EoS indicates that the chemical potential is calculated using an equation of state describing the polymer + solutes mixture. The equations for the free amorphous domains embody the assumption that the polymer behaves approximately like a subcooled polymer liquid in those regions, at least when it comes to swelling. On the other hand, the constraint pressure *P*_c_ appearing in the equations for the interlamellar domains formally reflects the compression of the interlamellar domains due to the presence of tie-molecules. As noted in our previous publication,^33^ the constraint pressure formalism is rigorously valid for any polymer system when the Helmholtz free energy can be written as the sum of the free energy of a subcooled polymer liquid *A*^EoS^ and a perturbation Δ*A*^c^ due to the formation of cross-links, which does not explicitly depend on the solute composition. This is indeed the approximate form of the free energy of the interlamellar domains in our model (*A*^IL^), where the crystals act as physical cross-links between the polymer chains. After a series of simplifying assumptions the constraint pressure can be expressed as^33^
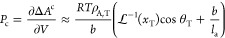
5where the partial derivative is taken at constant temperature and composition and assuming that the chain topology in the interlamellar domains is not altered during swelling. Here, *R* is the universal gas constant; *b* is the Khun length of the polymer;  is the inverse Langevin function; *x*_T_ and cos θ_T_ are the (average) fractional extension of the tie-molecules (i.e., the end-to-end distance divided by the maximum end-to-end distance) and the angle with respect to the normal to the lamellar surface, respectively; *l*_a_ is the interlamellar distance; and ρ_A,T_ is the average surface density of stems attached to tie-molecules on the lamellar surface. This latter quantity is central to the model and can be written as the product of the surface stem density ρ_A_ at the interface between the lamellae and the (interlamellar) amorphous domains, and the average fraction of stems, *p*_T_, that are connected to tie-molecules
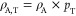
6

While no direct measurement of *p*_T_ is available,^19^ ρ_A_ can be estimated by considering the crystal structure of the polymer and calculating the average chain density in the plane perpendicular to the chain direction. For example, using the lattice parameters of the orthorhombic unit cell of crystalline PE,^63^ we obtain ρ_A_^PE^ ≈ 5.50 nm^–2^. This procedure neglects the chain tilt, i.e., the angle γ between the chain direction in the crystal and the normal to the interface between the lamellae and the interlamellar domains, which is usually in the range 20–40° in PE.^64,65^ Including the chain tilt would reduce ρ_A_ by a factor cos γ and therefore require higher values of *p*_T_ at fixed ρ_A,T_.

The local equilibrium hypothesis is incorporated in the model by imposing that the partial derivative of the Helmholtz free energy of the interlamellar domains *A*^IL^ with respect to the number of tie-molecule monomers *n*_T_ is equal to the driving force of crystallization μ_p,mono_, i.e., the chemical potential of the polymer per monomer in the crystalline lamellae
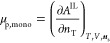
7Here, the derivative is taken at constant temperature, volume, and number of solute molecules in the IL domains (***n***_s_). Equation 7 allows one to estimate the equilibrium number of tie-monomers *n*_T_ (appearing implicitly on the right-hand side) as a function of μ_p,mono_ and the other thermodynamic variables. In our original model,^33^ μ_p,mono_ only accounts for bulk crystallization and is therefore approximated at each temperature and pressure with
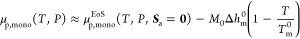
8where Δ*h*_m_^0^ and *T*_m_^0^ are the specific (i.e., by mass) enthalpy of melting and the melting temperature of a perfect polymer crystal and *M*_0_ is the molecular weight of the monomer. μ_p,mono_^EoS^(*T*,*P*,***S***_a_ = 0) is simply the chemical potential per monomer of a pure subcooled polymer—a quantity that is independent of the polymer’s molecular weight for long enough chains.^33^ More sophisticated models for μ_p,mono_ can be considered by including surface free energy effects (e.g., a Gibbs–Thomson term^66^ accounting for the finite thickness of the lamellae).

The solution of eqs 4 and 7 complemented by eq 8 yields the equilibrium number of tie-chain monomers *n*_T_ and the solubilities ***S***_a_^IL^ in the interlamellar domains at each temperature, pressure, and composition of the external fluid. Alternatively, as shown in ref (33), the solution can be more conveniently found via minimization of an appropriate thermodynamic potential. Critically, in contrast to the MH theory, knowledge of the equilibrium number of monomers allows us to predict the variations of the crystallinity of the lamellar stacks ω_c_^LS^ (defined as the ratio of the crystalline polymer mass and the sum of the inter-lamellar and crystalline polymer mass) due to temperature or swelling via
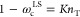
9

The constant *K* appearing in eq 9 can be found self-consistently after specifying the tie-molecule fraction *p*_T_ and the crystallinity at a given temperature.^33^eq 9 only holds if all the polymer chains in the interlamellar domains are tie-molecules, i.e., there are no unentangled loops or chain ends. This simplification does not change the qualitative features of the model as these types of chains do not contribute to the constraint pressure in the inter-lamellar domains.^33^ See the original work^33^ for a discussion on the impact of this approximation.

Finally, at each temperature *T*, pressure *P*, and external fluid composition ***y***, knowledge of ω_c_^LS^, ***S***_a_^IL^, and ***S***_a_^F^ allows for the calculation of the solubility of each component
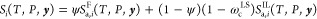
10

It should be noted that while ψ is a constant due to the simplified description of the free amorphous domains in the present model, the crystallinity of the lamellar stacks ω_c_^LS^ is a function of state (cf. eq 9). While the solubility in the free amorphous domains ***S***_a_^F^ is simply ***S***_a_^EoS^—i.e., the solubility in a subcooled polymer melt (cf. eq 4)—the solubility of each solute *i* in the interlamellar domains is lower due to the action of *P*_c_
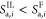
11

In particular, since larger values of *p*_T_ result in a higher constraint pressure (cf. eq 5), the solubility in the interlamellar domains of any given solute *i* decreases with increasing *p*_T_. To be precise, eq 11 holds only if the partial molar volume of the solute *i* in the polymer mixture is positive, a condition that is met for most nonionic fluids far from the critical point.

### 2.2 Molecular Models and Parameters

In the current model, a semicrystalline sample in contact with a fluid system is fully characterized by specifying an equation of state and related molecular parameters, a set of polymer-specific parameters (i.e., quantities that are uniform across all samples of a given polymer), and a set of sample-specific parameters.

#### 2.2.1 Equation of State Parameters: SAFT-γ Mie

An equation of state is necessary to calculate the Helmholtz free energy and chemical potentials appearing in eqs 4, 7, and 8. While any equation of state capable of describing polymer + solutes mixtures and pure fluids could be used (see, e.g., the Sanchez–Lacombe^67,68^ equation of state or PC-SAFT^69,70^), in the current work, the SAFT-γ Mie group-contribution equation of state^71,72^ is employed due to its success in describing both complex organic molecules and polymers. In SAFT-γ, Mie molecules are modeled as fully flexible heteronuclear chains of fused spherical segments. Each segment or group interacts with other segments via a 4-parameter Mie potential representing repulsion and dispersion forces; additionally, any segment can possess any number of association sites that mediate short-ranged directional interactions (i.e., hydrogen bonding) with compatible sites on other segments.^71,73,74^

In the current work, the sorption isotherms of various compounds in PE are calculated. Conveniently, the group-contribution basis of SAFT-γ Mie allows us to use a unique set of SAFT-γ Mie group parameters to represent the same functional group on different molecules.^75^ Methane is modeled as a single CH_4_ group. Linear *n*-alkanes are modeled as a sequence of *n* – 2 methylene groups (CH_2_) and two methyl groups (CH_3_). Branched alkanes are modeled with the aforementioned groups in addition to the ternary and quaternary carbon groups (CH and C, respectively). Linear alkenes are modeled with two methyl groups, a variable number of methylene groups and the sp^2^ carbon groups (CH_2_= and CH=). Finally, cyclohexane is modeled as six cyclic methylene groups cCH_2_ and benzene as six aromatic carbon groups aCH.

In all of the calculations presented here, PE is modeled as a linear homopolymer made of 1000 CH_2_ groups. The number of repeating units is arbitrary as the molecular weight of the polymer does not significantly affect the gas solubility or polymer density as long as the molecular weight is high enough.^33,42^ Furthermore, the end groups are not included as they are expected to have a negligible impact on the thermodynamic properties of long polymers. It is critical to ensure that the equation of state correctly represents the vapor–liquid equilibrium properties of each polymer + solute mixture before attempting to optimize any sample-specific parameters, as an inaccurate model at the EoS level can introduce systematic errors in the solubility predictions. All of the like and unlike SAFT-γ Mie parameters for these groups have been determined in previous work^72,76,77^ to capture pure component properties (such as saturation densities and vapor pressure) and mixture properties (such as fluid-phase boundaries and excess enthalpy of mixing) of linear alkanes and their mixtures.

**Figure 1:**
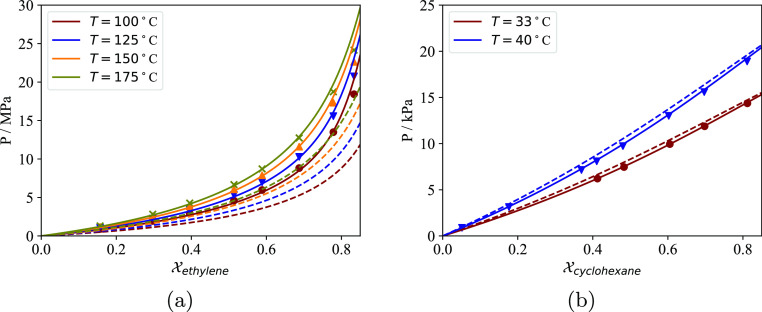
Bubble pressure curves of (a) ethylene + tetracontane and (b) cyclohexane + eicosane mixtures as a function of the composition of the solute. The continuous curves represent SAFT-γ Mie calculations using the newly proposed molecular models (i.e., Mie force-field parameters) for ethylene and cyclohexane (cf. Section 2.2.1); the dashed curves represent calculations using only SAFT-γ Mie parameters developed in previous work.^76^ Experimental data are shown as symbols and are taken from de Loos et al.^78^ and Gómez-Ibáñez et al.^79^

However, after comparison with literature data, we find that the solubility of ethylene (i.e., the bubble pressure curve) in long *n*-alkanes is overpredicted using the current parameter set (see Figure 1a), while the agreement is much better for mixtures of longer 1-alkenes and *n*-alkanes. Such a discrepancy is to be expected: ethylene comprises only two CH_2_= groups, whose parameters were optimized to reproduce the properties of the series of 1-alkenes.^76^ Since ethylene is the smallest representative of the series, the electronic environment around its two carbons can be expected to be noticeably different than that in longer alkenes, resulting in a different effective dispersion potential. In order to tune the least number of parameters, we define here the new second-order group CH_2_^eth^= to model ethylene. This group possesses the same like and unlike interaction parameters as its previously defined counterpart (CH_2_=), with the exception of the unlike dispersion energy , which is modified to reproduce the solubility of ethylene in tetracontane (Figure 1a). This ensures that this modification affects only the properties of ethylene-containing mixtures and not of all the other compounds containing the CH_2_ and CH_2_= groups. The optimal value for the dispersion energy was found to be 362.79 K, as opposed to the published value of 386.80 K.

At the same time, the solubility of cyclohexane in long *n*-alkanes is slightly under-predicted as noted in our previous work.^33^ The unlike interaction energy between the cCH_2_ and CH_2_ groups is therefore refined from 469.67 to 471.85 K, a value that more accurately reproduces cyclohexane solubility in eicosane (see Figure 1b).

#### 2.2.2 Polymer and Sample-Specific Parameters

The polymer-specific parameters are quantities like the specific enthalpy of melting of a perfect polymer crystal Δ*h*_m_^0^ that are uniform across all semicrystalline samples by definition, and can be readily found in the literature (see Table 1). On the other hand, each sample possesses a number of unique properties resulting from its crystallization history, molecular weight distribution, and branching content. In the present model, four parameters are used to uniquely characterize a semicrystalline sample:

- *p*_T_, the average fraction of crystalline stems connected to tie-molecules in the interlamellar domains;
- ψ, the mass fraction of free amorphous domains relative to the total polymer mass;
- ω_c_* = ω_c_(T*), the crystallinity of the pure sample at a given temperature T*;
- *l*_a_* = *l*_a_(T*), the average interlamellar distance of the pure sample at a given temperature T*.

**Table 1:** Polymer-Specific Parameters for PE Used for All of the Calculations^20,63,80,81^^a^

property	symbol	value
bond angle	θ_B_	109.47°
bond length	*l*	0.154 nm
enthalpy of melting	Δ*h*_m_^0^	293 J g^–1^
melting temperature	*T*_m_^0^	414 K
surface stem density	ρ_A_	5.50 nm^–2^
monomer molecular weight	*M*_0_	14.03 g mol^–1^
flory characteristic ratio	*C*_∞_	6.9

a The enthalpy and temperature of melting refer to the values for a perfect polymer crystal. The surface stem density is the chain density along the (001) plane of the orthorhombic unit cell of PE.^63^

The other thermodynamic properties of the sample that are relevant to the present model can be calculated once this parameter set is specified. It is necessary to specify the temperature T* at which the last two quantities are measured because the model allows one to predict their variation with temperature, in accordance with experimental evidence.^42,48^

When comparing the model predictions to experimental sorption isotherms, the value of the interlamellar distance at a specific temperature *l*_a_* is found to not influence the chemical potentials significantly and is therefore set to the typical value of 10 nm at 25 °C for PE^26,54^ without affecting the solubility calculations.^33^ Crystallinity, on the other hand, has a great impact on the calculations and must be measured for each sample with one of the techniques outlined at the beginning of Section 2.1.

This leaves *p*_T_ and ψ as the only free parameters of the model that are optimized to reproduce pure-component solubility data at a given temperature.

Estimating *p*_T_ and ψ at the same time can, however, lead to parameter degeneracy when the available solubility data for a given polymer sample includes measurements for only one solute in the low-pressure (Henry) regime. In this limit, the solubility must increase linearly with pressure due to the ideal gas behavior of the external fluid and Henry’s law for the polymer–solute mixture,
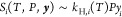
12as *P* → 0. Here, *k*_H,*i*_ is the Henry constant of solute *i* and *y*_*i*_ is its mole fraction in the external gas. At infinite dilution, our model (eq 10) predicts
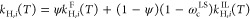
13and since the Henry constant in the interlamellar domains decreases with *p*_T_ and *k*_H,*i*_^F^ > *k*_H,*i*_^IL^ (as the interlamellar domains are constrained) an infinite number of pairs of *p*_T_ and ψ result in the same overall Henry constant for the solute *i*. For example, in our previous work,^33^ it was shown how the linear increase in solubility at low pressures can be described accurately both by setting ψ = 0 and only adjusting *p*_T_ or by adjusting both parameters at the same time.

Nonetheless, at pressures closer to the saturation point of the external fluid, the degeneracy disappears as the increase in solubility in the free amorphous domains is more pronounced than in the interlamellar domains. In particular, it is necessary to have ψ > 0 to reproduce the high solubilities near condensation as swelling in the interlamellar domains is severely restricted by the tie-molecules.^24,33^ Using solubility data of different solutes to parametrize the same sample can also help remove the degeneracy because the extent of solubility reduction in the interlamellar domains (compared to the free amorphous domains) is in principle different for each solute.

**Figure 2:**
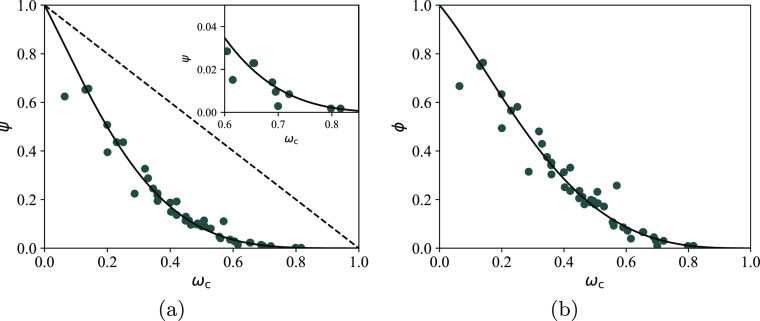
Fraction of free amorphous mass relative to the total polymer mass (ψ) and to the amorphous mass (ϕ) as a function of the crystallinity (ω_c_) of a semicrystalline PE sample. Symbols are experimental data at 25 °C from a variety of sources^61,82−88^ reported by Chmelař et al.;^61^ the continuous curve is an empirical correlation of the data (eq 14 with *C* = −0.3673); the dashed line in (a) represents the upper bound to ψ, i.e., 1 – ω_c_. The higher crystallinity data are shown in the inset for clarity.

In the current work, we adopt an ansatz for ψ as a function of the measured crystallinity in order to avoid degeneracy in the sample-specific parameters, as many of the sources considered (cf. Section 2.3) only reported solubility in the Henry regime or for a single solute. We remove ψ from the optimization following Chmelař et al.,^61^ who showed that the fraction of free amorphous mass can be estimated quite precisely using only crystallinity as an input by comparing measurement from difference sources and with different techniques (see Figure 6 of ref (61)). Here, we choose the function
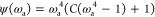
14to obtain ψ given ω_a_ = 1 – ω_c_, where *C* = −0.3673. This functional form ensures that ψ(0) = 0, ψ(1) = 1, which are physically sound constraints, selected among other low-order polynomials for its simple form and good accuracy in the entire crystallinity range. The value of *C* used is obtained by minimizing the mean-squared error between the predictions of eq 14 and the data reported by Chmelař et al., which refers to PE samples over a wide range of crystallinity analyzed using NMR,^61,82−86^ PALS,^87^ or a combination of DSC and WAXS.^88^ A comparison between calculations using eq 14 and experimental data is shown in Figure 2.

It is important to note that the experimental data used with eq 14 refer to PE samples at 25 °C and therefore should only be applicable to PE samples at the same temperature in the absence of a model detailing the variations of crystallinity or ψ with temperature. In practice, crystallinity measurements are sometimes made at temperatures different from room temperature (cf. Table 2). For simplicity, we estimate ψ for each sample based on the crystallinity at the reported temperature (ω_c_*) using eq 14.

**Table 2:** Crystallinity Characterization Techniques Employed for the Experimental Solubility Data in PE Considered in the Current Work^a^

source	technique	T*/°C
Chmelař et al.^36^	DSC, ρ	80
Doong and Ho^37^	ρ, DSC, SAXS	25
Santos et al.^89^	ρ	30
Jin et al.^90^	ρ	25
Kiparissides et al.^34^	DSC	25
Lopez-Gonzalez et al.^91^	ρ	30
Moebus and Greenhalgh^60^	Unknown	25
Moore and Wanke^38^	SAXS	25
Ben Mrad et al.^92^	DSC, ρ	70
Novak et al.^93^	DSC	25
Rausch et al.^58^	ρ	25
Sturm et al.^59^	ρ	25
Valsecchi et al.^33^	ρ	25
Von Solms et al.^94^	ρ	25
Yoon et al.^95^	ρ	25

a The quantity T* indicates the temperature at which the crystallinity measurement was made (cf. Table 3).

The only free parameter that we optimize to reproduce the sorption isotherms in the current work is therefore *p*_T_. Its value is found for each sample by minimizing the relative root mean squared error (RRMSE) between the model predictions and the experimental gas solubility at a given temperature *T*
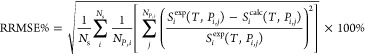
15Here, *N*_s_ is the number of single-solute isotherms used for each sample, *N*_*P*,*i*_ is the number of solubility measurements for each isotherm, and *P*_*i*,*j*_ is the pressure at each measurement. The calculated solubility *S*_*i*_^calc^ is implicitly a function of the polymer- and sample-specific parameters.

### 2.3 Sourcing Crystallinity and Solubility Data

Solubility is reported using different units across different sources; all experimental solubility data are here converted to g of solute per 100 g of pure polymer to aid comparison. The data are taken directly if reported in tables in the original publications or manually extracted from plots with PlotDigitizer.^96^ Unfortunately, in most cases, the uncertainty of the solubility measurements was not reported.

In order to compare data between different sources, it is also important to ensure that crystallinity is calculated using the same formulas and parameters consistently. The crystallinity estimated with density measurements is here recalculated using eq 1 and the correlation between crystalline and amorphous specific volumes of PE and temperature proposed by Chiang and Flory^20^
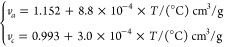
16Similarly, the crystallinity estimated via DSC analysis is rescaled using the specific enthalpy of melting of extended PE crystals reported in Table 1, Δ*h*_m_^0^ = 293 J/g.

## 3 Results and Discussion

### 3.1 Optimization Results

#### 3.1.1 Optimized Model Parameters for PE Samples

**Figure 3:**
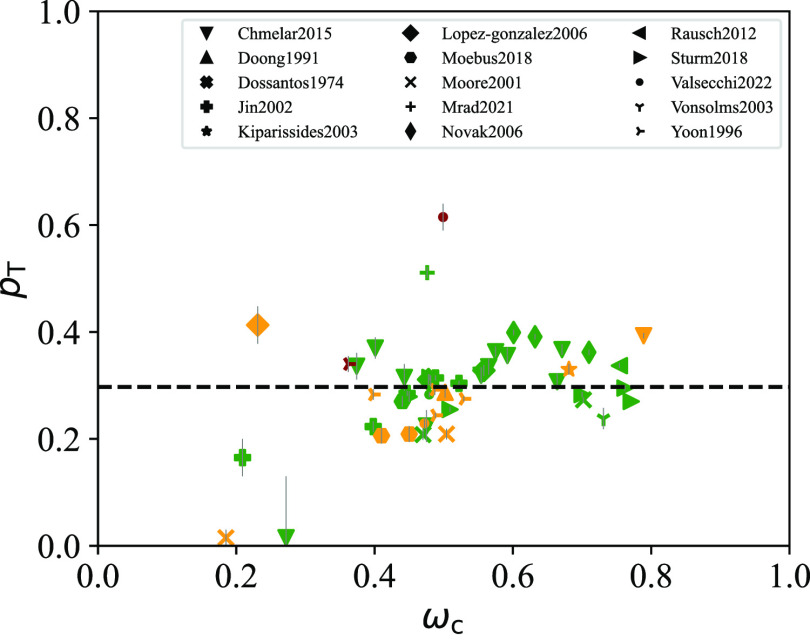
Optimal value of *p*_T_ for each PE sample considered as a function of the corresponding measured crystallinity ω_c_. Each symbol corresponds to a different literature source (see Table 3), while the color represents the RRMSE % at the optimum: green if 0% ≤ RRMSE % ≤ 10%, yellow if 10% < RRMSE % ≤ 30%, and red if RRMSE % > 30%. The horizontal dashed line indicates the average value of *p*_T_ (0.297) across all samples. The error bars indicate values of *p*_T_ that result in the RRMSE % being within 5% from the optimum. The solubility in the interlamellar domains of PE is negligible for *p*_T_ > 0.6. The temperature at which the crystallinity was measured was 25 °C for most samples (Table 2).

In Table 3, the results of the optimization of the polymer sample-specific parameters for all the semicrystalline PE samples considered are presented. For each sample, we report the temperature at which the solubility used in the parameter estimation was measured, the solutes considered, the crystallinity (measured at temperature T*), see Table 2, the source, and the denomination of the sample in the original publication. Furthermore, we report the optimal value of *p*_T_, the value of ψ calculated with eq 14, and the RRMSE at the optimum. In Figure 3 the optimal values of *p*_T_ are plotted as a function of the crystallinity of the sample. The average *p*_T_ value across all samples is 0.297, with an average RRMSE% of 8.43%. We consider this error to be acceptable, given the intrinsic uncertainty in the measurements of solubility and crystallinity.

**Table 3:** Model Parameters for the Semi-Crystalline PE Samples Considered^a^

ref	sample name	ω_c_*	*p*_T_	ψ	solutes	*T*/°C	RRMSE%
Chmelař et al.^36^	VLLDPE	0.272	0.015	0.728	C2=	80	0.68
	LLDPE A	0.374	0.336	0.201	C2=	80	2.61
	LLDPE B	0.401	0.370	0.170	C2=	80	1.02
	LLDPE C	0.443	0.315	0.128	C2=	80	5.22
	LLDPE D	0.475	0.224	0.102	C2=	80	2.32
	MDPE A	0.554	0.319	0.053	C2=	80	3.17
	MDPE B	0.565	0.335	0.048	C2=	80	1.26
	MDPE C	0.575	0.363	0.038	C2=	80	3.30
	HDPE A	0.592	0.356	0.017	C2=	80	1.21
	HDPE C	0.664	0.307	0.016	C2=	80	5.14
	HDPE D	0.671	0.367	0.010	C2=	80	1.90
	HDPE E	0.789	0.393	0.005	C2=	80	11.62
Doong and Ho^37^	PE film	0.502	0.287	0.083	aC6	30	14.62
Santos et al.^89^	DYND-3	0.481	0.308	0.097	C4, C4= , iC4	30	12.33
Jin et al.^90^	MTH879	0.209	0.165	0.479	C6=	50	8.17
	MTH904	0.398	0.223	0.173	C6=	50	4.29
	MTH912	0.447	0.285	0.125	C6=	50	5.18
	MTH918	0.488	0.314	0.092	C6=	50	3.13
	MTH923	0.522	0.304	0.070	C6=	50	3.19
Kiparissides et al.^34^	HDPE	0.681	0.330	0.014	C2=	80	11.62
Lopez-Gonzalez et al.^91^	LDPE87	0.231	0.413	0.433	C2=, C3=	30	20.6
	LDPE91	0.478	0.311	0.099	C2=, C3=	30	7.85
	LDPE93	0.558	0.328	0.052	C2=, C3=	30	7.20
Moebus and Greenhalgh^60^	EH1	0.410	0.206	0.160	C2=, iC4, iC5, C6, C6=	80	11.69
	EH5	0.440	0.270	0.131	C2=, iC4, C6, C6=	85	9.18
	EB1	0.450	0.209	0.122	iC4, C4= , iC5	85	13.15
Moore and Wanke^38^	LLDPE (cohexene)	0.185	0.015	0.532	C4=, C6=	69	12.80
	LLDPE (cobutene)	0.470	0.208	0.106	C4=, C6=	69	8.37
	LDPE	0.504	0.209	0.081	C2=, C4=, C6=	69	10.78
	HDPE	0.702	0.273	0.011	C4=, C6=	69	7.72
Mrad et al^92^	LDPE	0.476	0.511	0.101	C2=	70	7.81
Novak et al.^93^	HDPE—sample 1	0.601	0.399	0.034	C2=, C6=	70	4.62
	HDPE—sample 2	0.632	0.391	0.025	C2=, C6=	70	7.75
	HDPE—sample 3	0.710	0.362	0.010	C2=, C6=	70	4.64
Rausch et al.^58^	HDPE	0.754	0.337	0.005	cC6	90	3.26
Sturm et al.^59^	VLLDPE—sample 5	0.450	0.279	0.122	iC5, C6=	50	9.85
	LLDPE—sample 6	0.480	0.315	0.098	C6=	50	4.12
	LLDPE—sample 1	0.509	0.255	0.078	iC5	50	1.16
	HDPE—sample 3	0.700	0.283	0.011	iC5	65	1.70
	HDPE—sample 2	0.762	0.295	0.004	iC5	50	5.27
	HDPE—sample 4	0.771	0.270	0.004	iC5	50	3.91
Valsecchi et al.^33^	LDPE	0.472	0.229	0.104	C6, cC6, C7	25	11.26
	MDPE	0.479	0.283	0.099	C6, cC6, C7	25	5.98
	HDPE	0.499	0.621	0.085	C6, cC6, C7	25	36.53
Von Solms et al.^94^	HDPE	0.731	0.238	0.007	C1	25	2.93
Yoon et al.^95^	LLDPE—sample 1	0.362	0.340	0.216	C6=, C8=	70	39.89
	LLDPE—sample 2	0.398	0.283	0.173	C6=, C8=	70	12.42
	LLDPE—sample 3	0.487	0.292	0.093	C6=, C8=	70	14.36
	LLDPE—sample 4	0.488	0.244	0.092	C6=, C8=	70	17.58
	LLDPE—sample 5	0.529	0.275	0.066	C6=, C8=	70	14.74

a The crystallinity ω_c_* is measured at the temperature T* as reported in Table 2, and ψ is calculated using eq 14. The solutes used to parametrize each sample are reported in the “solutes” column: C*n* refers to *n*-alkanes; C*n*= to linear alk-1-enes; iC4 and iC5 to isobutane and isopentane, respectively; and cC6 for cyclohexane; and aC6 to benzene. *T* is the temperature of the sorption isotherms used for each sample, and the RRMSE is the minimum relative root mean squared error calculated with eq 15.

There is no direct experimental evidence for the value of *p*_T_ in semicrystalline polymers. Of all the polymer stems emanating from a lamella, a fraction *p*_TF_ performs tight-folds back into the crystal, meaning that they are connected to loops that only span the interface between the lamella and the interlamellar amorphous domain—a region of dimension which is of the order of the Khun length of the polymer.^64,65^ Through theoretical calculations and Monte Carlo simulation, it has been argued that the fraction of tight-folds should lie in the range 0.6–0.8 for flexible polymers like PE.^64,65,97−99^ At the same time, the inequality *p*_T_ < 1 – *p*_TF_ must hold as some stems on the lamellar surface could be connected to tails or unentangled loops, which are not tie-molecules. The typical value of *p*_T_ ∼ 0.3 found for the PE samples considered not only conforms to these bounds but also seems to suggest that all the interlamellar amorphous mass is connected to tie-molecules (i.e., bridges and entangled loops) as *p*_T_ + *p*_TF_ ≈ 1. This is consistent with the simplifying assumption employed in the model of neglecting unentangled loops and tails.^33^ Furthermore, a high content of tie-molecules in the interlamellar domains is to be expected due to defect segregation in these domains during crystallization.^100,101^

Note that both the reported bounds for *p*_T_ and our present model assume that there is no chain tilt,^33,64,65,97^ i.e., that the stems are normal to the lamellar surface. In PE, the chain tilt is between ∼20 and 40°, which leads to a reduction in the amount of tight folds predicted in the aforementioned studies.^64,65^ As mentioned in Section 2.1, the optimal value of *p*_T_ needed to represent the experimental data with our model increases for nonzero tilt angles,^33^ suggesting that our results would predict the prevalence of tie-molecules in the interlamellar domains (i.e., *p*_T_ + *p*_TF_ ≈ 1) even if realistic chain tilts were considered.

Various authors have argued that there could be a maximum in the tie-molecule content at intermediate crystallinity based on mechanical measurements, sorption data, and theoretical calculations.^31,32,59,102^ Due to the scatter in our optimal *p*_T_ values when plotted against crystallinity, we cannot confirm this hypothesis unless more experimental data at high crystallinity are analyzed with the present model. Nevertheless, it can be argued that the crystallinity simply does not capture enough of the history of a sample to show a strong correlation with *p*_T_. For example, samples with same crystallinity but different average lamellar thickness should possess different tie-molecule fractions.^31,103^ For accurate estimates of *p*_T_, it may thus be necessary to consider additional properties for each PE sample such as its molecular weight distribution, branching content, and production history.

Finally, we note that systematic errors in the reported values of crystallinity and solubility can affect the comparison of data between different sources. For example, the LDPE sample studied by Mrad et al.^92^ and the HDPE sample reported in our previous work^33^ are clear outliers with *p*_T_ > 0.5 at ω_c_ ≈ 0.5. This can lead to the suggestion that using crystallinity (or density) alone to estimate a priori ψ and *p*_T_ at the same time can result in unphysical values of some of the parameters.

#### 3.1.2 Henry Constants of Ethylene

**Figure 4:**
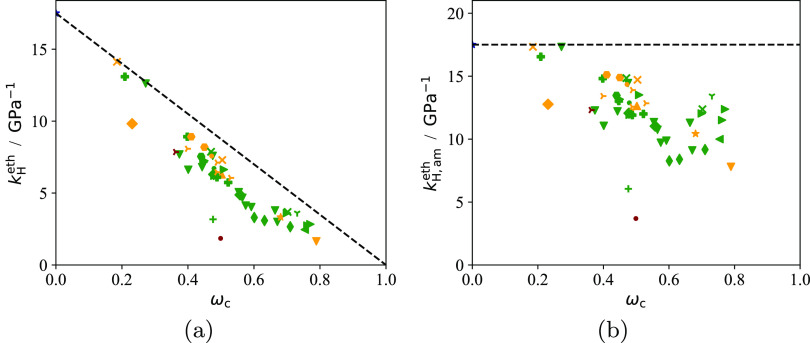
Henry constant of ethylene in the semicrystalline PE samples studied at 25 °C calculated using our current model and the optimized sample-specific parameters (Table 3) in (g/g) GPa^–1^ (see eq 13 for the definition). Symbols represent the calculations with the model after optimization of the sample-specific parameters of each sample (see Figure 3). The dashed lines correspond to predictions with ψ = 1 – ω_c_, i.e., with no constraints acting on the amorphous domains. (a) Henry constant per total polymer mass. (b) Henry constant per amorphous polymer mass.

In Figure 4 the Henry constant *k*_H_^eth^ (in (g/g)/GPa) of ethylene at 25 °C in each PE sample considered is plotted as a function of the crystallinity of the sample. It is particularly useful to plot the calculated value of the Henry constant using the optimized model parameters instead of the experimental value since the data are not always present or smooth enough in the low-pressure regime. Ethylene is chosen here as a “probe molecule” due to its importance in PE production processes. As expected, the Henry constant per total polymer mass decreases with increasing crystallinity (Figure 4a), always remaining below the ψ = (1 – ω_c_) line (i.e., the Michaels and Bixler model^104^). The Henry constant in the amorphous domains (intended as the sum of free and interlamellar domains) *k*_H,am_^eth^ = *k*_H_^eth^/(1 – ω_c_) is plotted in Figure 4b as a function of crystallinity of each sample. This quantity is seen to decrease on average with crystallinity and tends to the value predicted for a subcooled PE melt as ω_c_ → 0.

We note a greater (absolute) scatter in the data at high crystallinity in Figure 4b compared with Figure 4a; this phenomenon is likely due to the presence of the factor (1 – ω_c_) in the definition of *k*_H,am_^eth^, which therefore suffers from uncertainties in the measurements of the crystallinity ω_c_ when the latter is high. It is possible that a minimum in *k*_H,am_^eth^ (corresponding to a maximum in *p*_T_, cf. Figure 3) occurs at ω_c_ ∼ 0.6. Due to the scatter in the data, we believe that more measurements at ω_c_ ≥ 0.5 are needed to confirm or disprove this finding.

#### 3.1.3 Comparison with Data Included in the Parametrization

**Figure 5:**
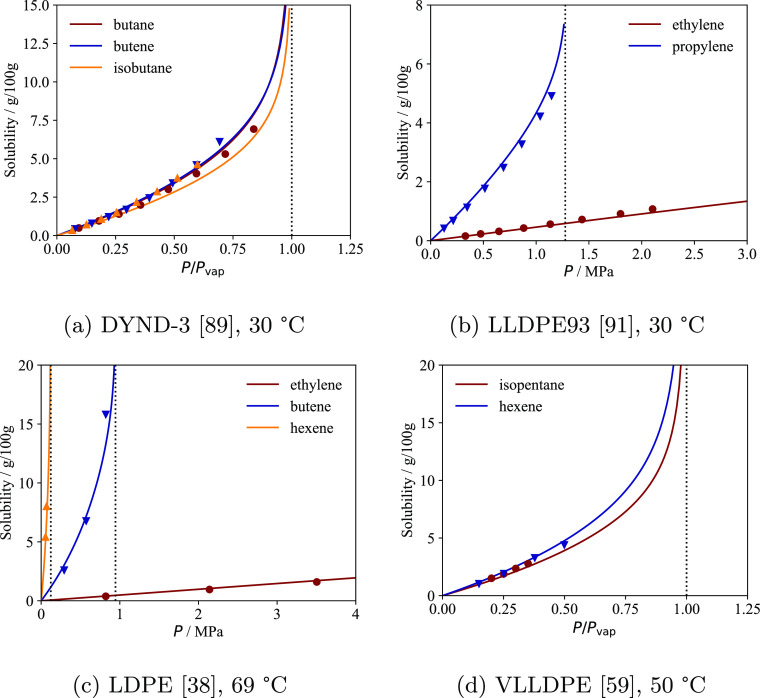
Solubility of various pure substances in semicrystalline PE samples analyzed in the literature. The continuous curves represent the calculations with the model with the sample-specific parameters reported in Table 3; for each sample, *p*_T_ is optimized to reproduce the experimental data (symbols). Vertical dotted lines represent the vapor pressure of the gas at each temperature. The solubility is plotted as a function of total pressure if one of the solutes is supercritical at the temperature considered; otherwise, the plots are represented as a function of the ratio between pressure and the vapor pressure of each solute at that temperature.

**Figure 6:**
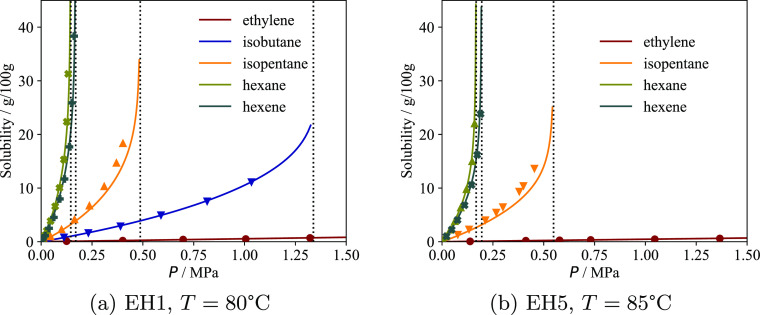
Solubility of various pure substances in semicrystalline PE samples analyzed by Moebus and Greenhalgh^60^ as a function of pressure. The continuous curves represent the calculations with the model, and the symbols represent experimental data. All isotherms are plotted up to the vapor pressure of the pure fluid at the corresponding temperature, with the exception of ethylene (which is supercritical at both temperatures). The *p*_T_ parameters of the two samples (0.206 and 0.270, respectively, cf. Table 3) are optimized to reproduce these sorption isotherms. (a) Solubility of pure fluids in the EH1 sample at 80 °C. (b) Solubility of pure fluids in the EH5 sample at 85 °C.

In Figures 5 and 6 the solubility calculations with the model are compared to experimental data for six PE samples for which sorption isotherms of multiple pure substances are available. The *p*_T_ parameter of each sample is optimized to reproduce all of the isotherms at the same time. Our calculations are in excellent agreement with the experimental data, confirming that a single parameter set can be assigned to each sample to capture the solubility of different pure substances. This demonstrates that the sample-specific properties of each semicrystalline PE sample can be effectively decoupled from the underlying equilibrium EoS, as shown in our previous work.^33^

### 3.2 Prediction of Cosolubility Effects

The robustness of the model is showcased by predicting cosolubility effects in a subset of the PE samples analyzed. The term “cosolubility effect” refers to the increase or decrease in solubility of a given compound in a sample due to the presence of other substances in the external fluid. This phenomenon is of critical importance in the production of polyolephines, as the addition of induced condensing agents like *n*-hexane or comonomers like 1-butene and 1-hexene to an ethylene reaction mixture has been shown to increase the polymerization rate of PE, presumably due to the increased ethylene solubility in the amorphous polymer grain near the catalyst sites.^60,92,93,105^ Furthermore, in real-world applications, semicrystalline polymers are rarely in contact with pure fluids, and factors such as the relative humidity can have an impact on the solubility of any given substance.

**Figure 7:**
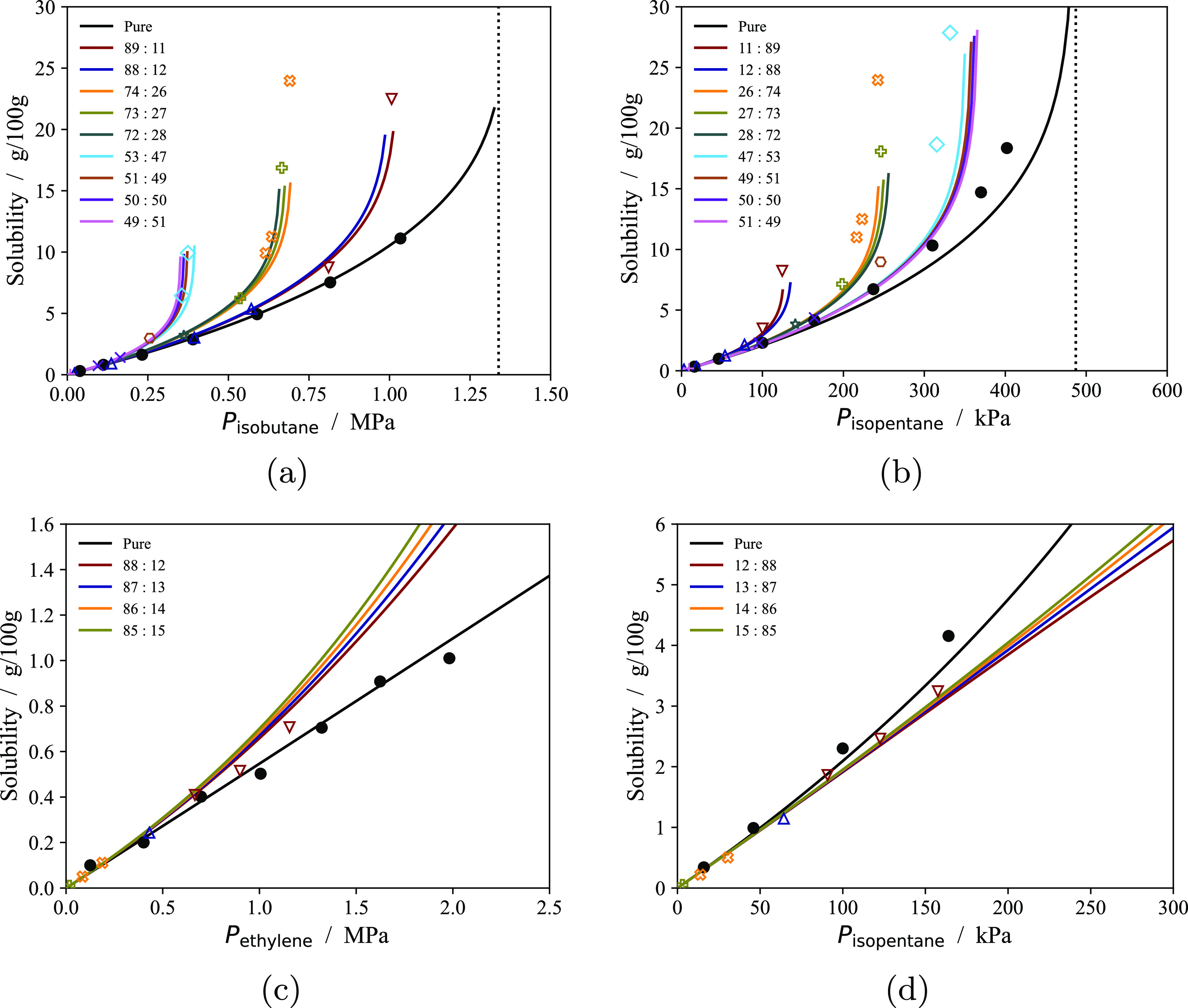
Solubility of individual components (in grams of solute *i* per 100 g of polymer) of fluid mixtures at 80 °C in contact with the EH1 sample analyzed by Moebus and Greenhalgh^60^ as a function of the corresponding partial pressure. The continuous curves correspond to predictions with our current model, and symbols represent the experimental data (color-coded with the corresponding curves). *p*_T_ is adjusted to reproduce the pure component data (black curves). The numbers in the legend refer to the composition of the two components (in % mol) in the external mixture. Vertical dotted lines, if present, indicate the vapor pressure of the pure fluids at 80 °C. (a,b) Solubility of isobutane (isopentane) in the sample at varying isopentane (isobutane) concentrations of an isobutane-isopentane mixture. (c,d) Solubility of ethylene (isopentane) in the sample at varying isopentane (ethylene) concentrations of an ethylene-isopentane mixture.

**Figure 8:**
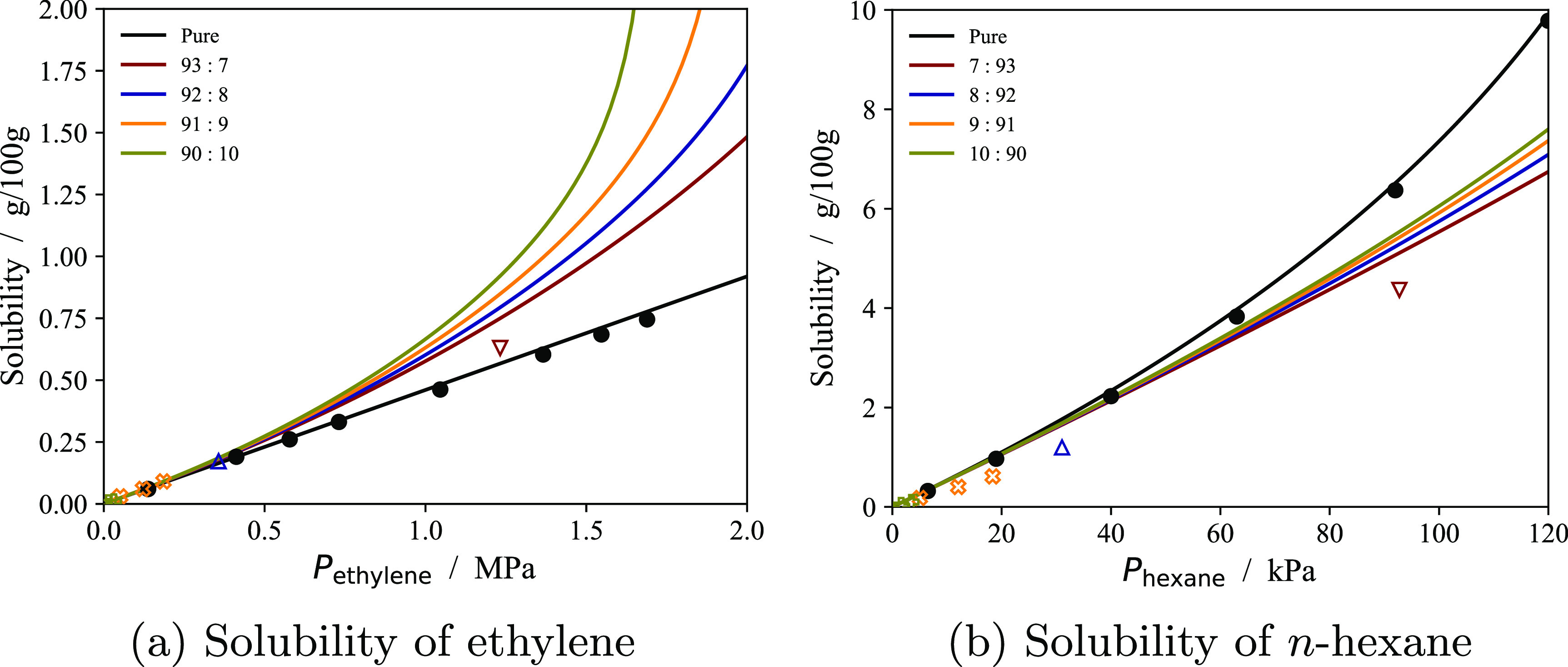
Solubility of individual components (in g of solute *i* per 100 g of polymer) of mixtures of ethylene and *n*-hexane at 85 °C in the EH5 sample analyzed by Moebus and Greenhalgh^60^ as a function of the corresponding partial pressure. The continuous curves correspond to predictions with our current model, and symbols represent the experimental data (color-coded with the corresponding curves). The numbers in the legend refer to the % mole fraction of the two components in the external mixture.

Our model naturally allows us to predict the solubility of mixtures in contact with a semicrystalline polymer, as outlined in Section 2.1. The SAFT-γ Mie equation of state used in the current work provides a good description of mixture properties as its parameters are usually optimized to reproduce the enthalpy of mixing and/or vapor–liquid equilibrium envelopes of fluid mixtures.^72,75,77,106−109^ In Figures 7 and 8 experimental cosolubility data of various substances in semicrystalline PE samples reported by Moebus and Greenhalgh^60^ are compared to the predictions with the model. Note that the optimal *p*_T_ parameter for each sample (Table 3) has been adjusted to reproduce the single–solute isotherms reported in Figure 6, whereas the values of ψ are estimated using eq 14.

The model semiquantitatively predicts the solubility of individual components of isobutane + isopentane and ethylene + isopentane mixtures in the EH1 sample (Figure 7) and of ethylene + *n*-hexane mixtures in the EH5 sample (Figure 8). As expected, the solubility of a component is only a function of its partial pressure *P*_*i*_ (the product of the total pressure *P* and its mole fraction in fluid *y*_*i*_) at low partial pressures. However, at higher partial pressure different mixtures display one of two types of behavior.

In the isobutane + isopentane mixture, the solubility of either component at fixed partial pressure is greatly enhanced by the presence of the other component in the external fluid. This phenomenon can be rationalized by realizing that solubility generally increases the most near saturation conditions of the external fluid (see Figure 6). All of the isotherms in Figure 7a,b are calculated up to the dew pressure of the mixture at each composition. The absolute value of the dew pressure of the isobutane + isopentane mixture is not influenced significantly by composition (due to the similarity of the saturation pressure of the two pure fluids), and therefore the partial pressure at condensation of each component is lowered as the corresponding composition in the external mixture is lowered. Isotherms for isopentane are systematically overpredicted as in Figure 6; this is likely a consequence of the overestimation of the vapor pressure of isopentane with the SAFT-γ Mie parameters in use.^106^

Conversely, in the case of both ethylene + isopentane and of ethylene + *n*-hexane mixtures (Figures 7c,d, and 8), the solubility of the lighter component (i.e., ethylene) is enhanced by the presence of the heavier component (i.e., isopentane or *n*-hexane), while the contrary is true for the solubility of the heavier components upon increasing the concentration of ethylene. According to our calculations, this phenomenon is so significant that at fixed temperature and total pressure, the calculated solubility of ethylene is greater if the external fluid is a mixture instead of pure ethylene, as can be seen in the figures.

A positive, albeit more modest, deviation of the solubility of ethylene from Henry’s law is also seen in the experimental data. Nevertheless, solubility measurements at higher pressures are needed to test the predictions outside the dilute regime. A systematic overprediction of the *n*-hexane solubility when mixed with ethylene is also observed, although we note the unusual behavior of the experimental Henry constant reported for *n*-hexane, which appears to change with the external composition.^60^

**Figure 9:**
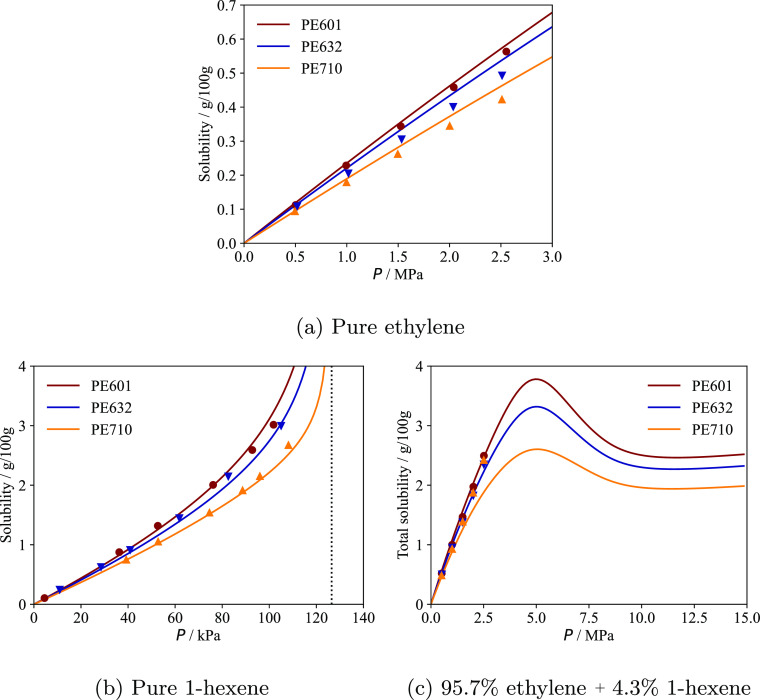
Solubility of ethylene, 1-hexene, and total solubility of a 95.7% ethylene + 4.3% 1-hexene mixture (mol %) in the semicrystalline PE samples analyzed by Novak et al.^93^ at 70 °C as a function of pressure. The continuous curves represent the calculations, and the symbols represent the experimental data. The *p*_T_ parameters of the three polymer samples are optimized to reproduce the respective pure component isotherms (see Table 3). The numbers next to PE in the legend refer to the crystallinity ω_c_ at 25 °C of the samples in parts per thousands.

Novak et al.^93^ reported measurements of the total solubility (i.e., the sum of the solubilities of each component) of ethylene +1-hexene mixtures in three HDPE samples. In Figure 9 pure component and mixture solubility data in the three samples are compared with the predictions of our current model. The *p*_T_ parameters (cf. Table 3) are optimized to provide a quantitative description of the pure component isotherms (Figure 9a,b), although the solubility of ethylene is slightly overestimated. The predicted total solubility of a 95.7% ethylene + 4.3% 1-hexene mixture (mol %) in the three samples is in good agreement with the experimental data, with the exception of the sample with the highest crystallinity (PE732). Interestingly, the discrepancy appears to be due to the model predicting a decrease in solubility with an increasing pressure between 5 and 10 MPa for all samples.

**Figure 10:**
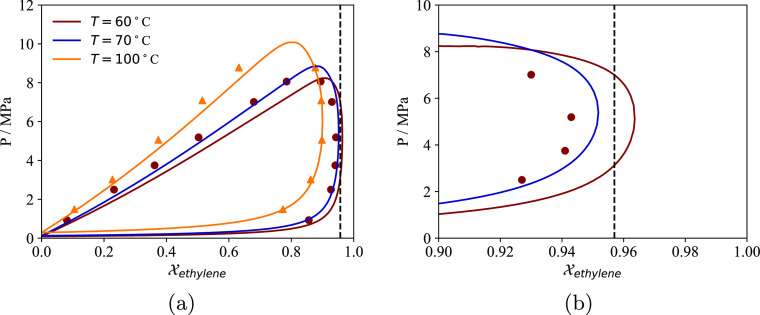
Isothermal VLE envelope of the ethylene +1-hexene mixture. The continuous curves are predictions using SAFT-γ Mie. The symbols represent experimental data from Laugier et al.^110^ The vertical dotted line corresponds to the composition of the mixture analyzed by Novak et al.^93^ i.e. *x*_ethylene_ = 0.957. (a) VLE envelope (b) VLE envelope for 0.9 < *x*_ethylene_ < 1.

This artifact in the prediction is due to our SAFT-γ Mie model predicting a critical composition of 95.2% for the ethylene + 1-hexene mixture at 70 °C, which is slightly below the composition of the mixture considered by Novak et al. (Figure 10). This causes the calculated partial molar volume of ethylene in the mixture to be negative for total pressures between about 5 and 10 MPa and leads to a decrease in the the total solubility with increasing pressure over the same range (Figure 9c). The actual critical composition of the mixture must be lower than the critical composition at 60 °C, ∼94.3%, as seen in the experimental data by Laugier et al. in Figure 10. Since, in reality, the mixture analyzed by Novak et al. is farther away from the two-phase region of the VLE envelope, the measured total solubility data thus displays a regular Henry behavior. This artifact in the predictions highlights the importance of using good molecular models for the fluids studied; it is likely that an SAFT model finely optimized for ethylene would result in very linear sorption isotherms for the mixture composition studied.

### 3.3 Solubility Predictions at Different Temperatures

In the present model, both *p*_T_ and ψ are independent of temperature and composition: the former because the topology of the interlamellar domains should not change unless the temperature is close to the melting point, where structural reorganization following recrystallization may occur, and the latter due to the simplicity of the model for the free amorphous domains, which are assumed not to exchange mass with the lamellae. Since the value of *p*_T_ for each sample is adjusted to reproduce sorption isotherms at a single temperature and ψ is estimated via eq 14, it is important to test the predictions of the model at temperatures different from the one at which the sample-specific parameters have been estimated.

**Figure 11:**
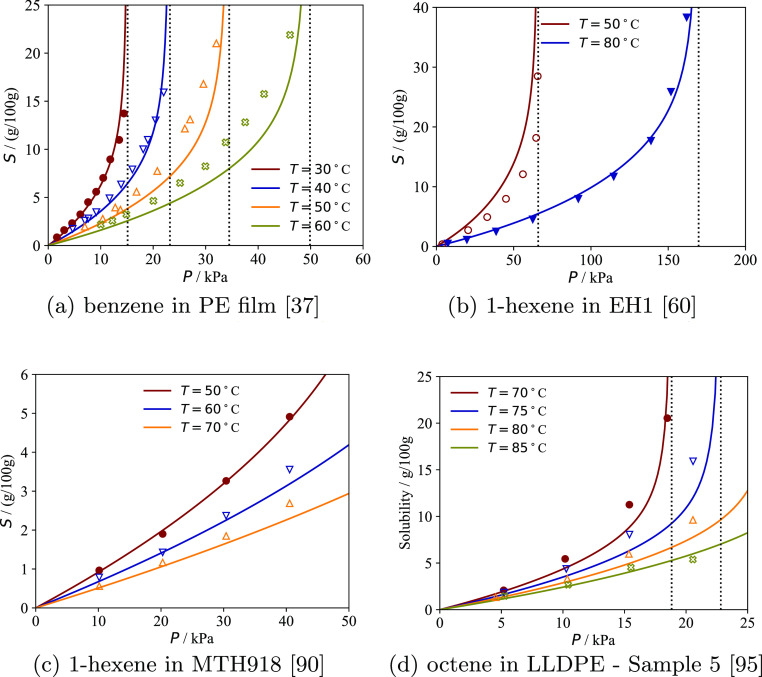
Solubility of various pure substances in semicrystalline PE samples at different temperatures using the optimal sample parameters reported in Table 3. The continuous curves represent the calculations with the model, and the symbols the experimental data. Data points represented by filled symbols are included in the parametrization procedure; the empty symbols are not included. Vertical dotted lines represent the vapor pressure of the external gas at each temperature.

**Figure 12:**
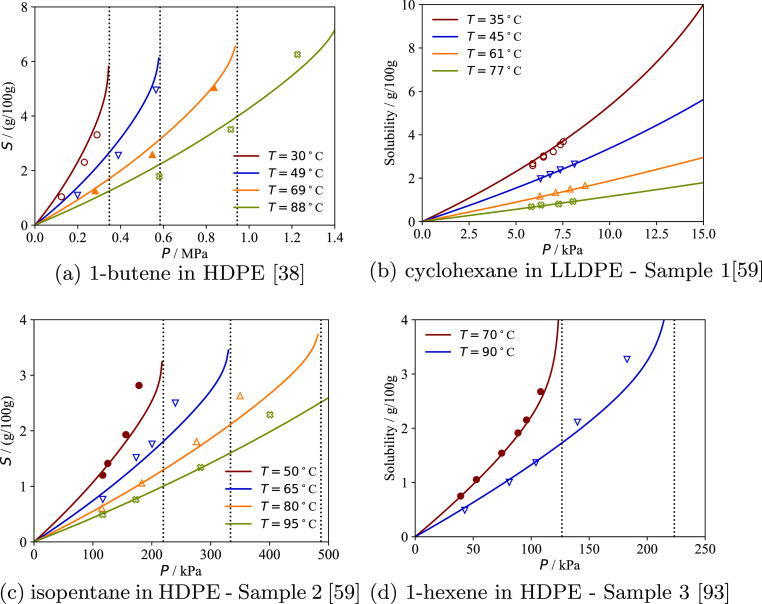
Solubility of various pure substances in semicrystalline PE samples at different temperatures using the optimal sample parameters reported in Table 3. The continuous curves represent the calculations with the model, and the symbols the experimental data. Data points represented by filled symbols are included in the parametrization procedure; the empty symbols, not included. Vertical dotted lines represent the vapor pressure of the external gas at the corresponding temperature.

In Figures 11 and 12 solubility predictions with the model at temperatures different from the ones included in the parametrization procedure are compared to experimental solubility data of pure substances in a subset of polymer samples. Overall, the calculations are in very good agreement with the data at all temperatures reported. It is particularly noteworthy that the solubility of cyclohexane in an LLDPE sample analyzed by Sturm et al.^59^ (Figure 12b) is accurately predicted at various temperatures when cyclohexane was not included in the parameter estimation procedure (cf. Table 3).

In general, the quality of the predictions deteriorates close to saturation of the external gas. This could be due to irreversible melting and reorganization of the lamellae, a phenomenon known to occur during swelling of semicrystalline PE at high solute activity.^28^ Since irreversible transformations result in changes to the crystallinity and microstructure of a polymer sample, these effects, if present, lead to hysteresis of the sorption/desorption cycle, which can be used to assess the degree of reversibility of the sorption process. However, desorption runs are rarely reported experimentally, preventing direct investigation of these effects here.

In some of the samples with lower crystallinity (Figure 11a–c), greater variations of solubility with temperature are predicted with the model than indicated by the experimental data. In the absence of irreversible transformations, these findings could indicate partial melting at the lateral lamellar surfaces^111^ leading to an increase in ψ with temperature. It may therefore be necessary to develop models to account for the changes of ψ with temperature to characterize semicrystalline polymers with temperature-independent sample-specific parameters over wide temperature ranges.

## 4 Conclusions

We have conducted a meta-analysis of semicrystalline PE samples investigated in the literature by employing a recently developed theoretical framework detailing the influence of tie-molecules and microstructure on the fluid solubility in semicrystalline polymers.^33^ Three parameters are assigned to each polymer sample to reproduce experimental solubility isotherms of pure hydrocarbons at temperatures below the melting point. The crystallinity parameter ω_c_ of a sample at a given temperature is calculated using density, DSC, or SAXS data reported in the literature. The fraction of free amorphous polymer mass ψ is estimated from the crystallinity using an empirical correlation (eq 14) based on a collection of experimental data reported by Chmelař et al.^61^ The average fraction of stems on the lamellae connected to tie-molecules *p*_T_ is then adjusted to capture the solubility of one or more pure components in the sample at a given temperature.

The average value of *p*_T_ across all samples (0.297, or ∼30%) conforms to bounds suggested by theoretical calculations and Monte Carlo simulation,^65,98−100^ and is consistent with one of the hypotheses underlying the model that all of the interlamellar polymer mass is connected to tie-molecules (bridges or entangled loops). The scatter seen in the optimal *p*_T_ values (cf. Figure 3) suggests that factors other than crystallinity (such as the molecular weight, comonomer content, and crystallization conditions of the polymer sample^19,31,32,103^) play a role in determining the chain topology in the interlamellar domains.

We believe that experiments combining solubility measurements of multiple substances and advanced sample characterization (such as low-field ^1^H NMR^61^ or small-angle neutron scattering^24,28^) are needed to determine the physical consistency of these assumptions and potentially uncover more compelling trends in the optimized parameters. Similarly, computer simulation of the interlamellar domains (e.g., Monte Carlo or molecular dynamics) is needed to assess the validity of the predictions with the model concerning both the chain topology and the stress state in these domains.

The calculations are found to be in very good agreement with the experimental pure-component solubility data, with an average RRMSE% of 8.43%. Overall, we demonstrate that the a single pair of *p*_T_, ψ parameters can provide an accurate representation of sorption isotherms of several compounds in the same PE sample (see, e.g., Figures 5 and 6), supporting the assumption that we can effectively decouple the sample-specific features of each semicrystalline PE sample from the intermolecular interactions between the polymer and each solute—which are here described accurately by the SAFT-γ Mie EoS.

In Section 3.2 cosolubility effects in semicrystalline PE samples are predicted using the optimized sample-specific parameters. For isobutane + isopentane mixtures, the solubility of either component at constant partial pressure is enhanced when mixed, compared to the pure case. Conversely, in mixtures of ethylene + isopentane, ethylene + *n*-hexane, and ethylene +1-hexene, the presence of the heavier component is found to increase the solubility of ethylene at a fixed partial pressure. We show that even moderate amounts of *n*-hexane (10% mol) mixed with ethylene could lead to a 2-fold increase in solubility of ethylene at partial pressures of ∼15 bar (total pressure ≈16.7 bar), which is just slightly above the typical range of operating pressures of fluidized bed reactors used in PE polymerization.^7,112^ We thus encourage experimental investigation at higher pressure, as the experimental data reported did not show significant variations from the Henry dilute regime.

The assumption that *p*_T_ and ψ are temperature-independent parameters is tested by comparing the model predictions with experimental sorption isotherms at temperatures different from the ones at which the optimal parameters are determined. Overall, the model predictions are found to be in very good agreement with the experimental data (Figures 11 and 12). Discrepancies between data and calculations are most evident in samples of low crystallinity at pressures close to saturation of the external fluid or at high temperatures, possibly due to irreversible transformations in the samples or inadequacies of the equation of state. Alternatively, it may be necessary to consider reversible changes in ψ with temperature or during sorption, which could occur in the presence of mass exchange at the lateral lamellar surfaces.^111^

Aside from the assumptions inherent in our approach, the proposed methodology is limited by the availability of experimental data. It is crucial that the crystallinity used for the calculations reflects closely the experimental value to avoid errors in the parametrization procedure—as exemplified by the poor performance of our model with the HDPE sample studied in our previous work^33^ and the LDPE sample of Mrad et al.^92^ (see Section 3.1.1, Section 3.2, and Figure 3). Furthermore, it would be preferable to compare only solubility data at the same temperature and with the crystallinity measured with the same method to minimize the likelihood of systematic errors in the experimental data.

## Data Availability

Data underlying this article can be accessed on Zenodo at 10.5281/zenodo.8308259, and used under the Creative Commons Attribution license.
